# Investigation of the knowledge of safeguarding in sports among medical and paramedical professionals in Rugby Africa, at Mauritius 7s 2024

**DOI:** 10.17159/2078-516X/2026/v38i1a24286

**Published:** 2026-09-15

**Authors:** A Bholah, J Somanah, M Putteeraj, N Sewry, HB Baptiste, L Gordon, M Hlávek, D Garnett

**Affiliations:** 1 Faculty of Medicine and Health Sciences, University of Mauritius, Reduit, Republic of Mauritius; 2 Mauritius Rugby Federation, Republic of Mauritius; 3 School of Health Sciences, University of Technology Mauritius, Port Louis, Republic of Mauritius; 4 Sport, Exercise Medicine & Lifestyle Institute (SEMLI), Faculty of Health Sciences, University of Pretoria, South Africa; International Olympic Committee (IOC) Research Centre of South Africa, Moka, Mauritius; 5 Sports and Exercise Medicine Association, Les Allees d’Helvetia, Moka, Mauritius; 6 Division of Physiological Sciences and Health through Physical Activity, Lifestyle and Sport Research Centre, Department of Human Biology, Faculty of Health Sciences, University of Cape Town, Cape Town, South Africa; 7 Faculty of Sports Science and Health, Matej Bel, University in Banská Bystrica, Tajovského, Slovakia; 8 Centre for Movement, Occupation and Rehabilitation Sciences (MOReS), School of Sport, Nutrition and Allied Health Professions Faculty of Health, Science and Technology, Oxford Brookes University, Oxford, UK

**Keywords:** Safeguarding, athletes, abuse, awareness, reporting, education, rugby

## Abstract

**Background:**

Safeguarding in sports has emerged as a critical issue globally, particularly for elite athletes in high-risk environments. Despite the presence of operationalised policy frameworks for safeguarding athletes from injury, violence, or harm, limited evidence exists on how well frontline medical professionals in Africa implement these practices. This study, therefore, investigates the extent of safeguarding knowledge, the degree of professional readiness, and the barriers to reporting among medical and paramedical personnel across Rugby Africa.

**Methods:**

A cross-sectional study was conducted at the 2024 Rugby Africa Men’s 7s in Mauritius. This study involved 19 Immediate Care in Rugby Level 2-certified medical and paramedical professionals from 11 African countries. Data were collected using a modified version of the Safeguarding Officer Pulse Survey, incorporating both quantitative and qualitative items. Descriptive statistics and thematic analysis were applied to assess knowledge, confidence, procedural awareness, and perceived organisational support regarding safeguarding responsibilities.

**Results:**

Over half (53%) of the respondents reported familiarity with safeguarding policies, yet only a small proportion (21%) had received formal training on how to apply those policies in practice. Confidence in identifying abuse was fair (63%), but awareness of designated safeguarding officers and reporting procedures was low. Environmental factors such as fear of retaliation (69%) and cultural silence (68%) were major barriers to effective reporting of safeguarding. Participants identified themselves (89%) as key protectors of athlete welfare, but lacked procedural clarity. Gaps in organisational education, policy implementation, and surveillance measures suggest systemic weaknesses in safeguarding infrastructure across Rugby Africa medical teams.

**Conclusion:**

Enhancing safeguarding awareness, procedural training, and cultural shifts are essential for creating a safe sporting environment for African Rugby. Structured education and transparent reporting frameworks are recommended for similar tournament contexts. Additional multi-country research is necessary to guide broader policy decisions.

Creating a safe, secure environment for engagement in sports is vital for athletes to excel and perform at their best. Safeguarding the well-being of athletes has emerged as a critical priority in the sports industry, particularly at World Rugby and Rugby Africa. Given the growing awareness of mental health challenges, injury risks, and maltreatment in this group, safeguarding is no longer a peripheral concern but a fundamental part of athlete welfare. The development of the Olympic Movement Medical Code and guiding statements from the International Olympic Committee Medical Commission were pivotal in defining sport safeguarding.^[1]^ While the code primarily addresses health protection, its principles inherently support the role of medical and paramedical professionals in identifying and responding to practices that may compromise athlete welfare, including potential violence or abuse. The Violence Prevention Alliance of the World Health Organisation defines the problem of violence as the “intentional use of physical force or power, threatened or actual, against oneself, another person, or against a group or community, that either results in or has a high likelihood of resulting in injury, death, psychological harm, maldevelopment or deprivation”.^[2]^ This definition, however, fails to address actions and behaviour that are construed as violent. In sports, abuse is often dismissed as motivational coaching.

A general increased interest in safeguarding has emerged over the past decades on account of revelations of the occurrence of malpractice, medical mismanagement, cyberbullying and sexual abuse towards individuals in sporting communities.^[3,4,5]^ Such studies have shed light on an alarming number of athletes who are victims of physical violence, emotional abuse and bullying from their coaches, with many studies also reporting cyberbullying from spectators or peers. The findings of Fasting, Huffman and Sand also revealed a concerning pattern of deliberate suppression of reporting through financial incentives.^[6]^ The shocking accounts of elite athletes have called attention to the systemic barriers of reporting to the IOC and the possible lack of supportive resources. The existing protection frameworks for vulnerable athletes embedded within established power hierarchies suffer from insufficient visibility and awareness.^[7]^ Athlete abuse, in any form, is unacceptable. Effective recognition and reporting procedures are needed to relieve the burden on athletes and place accountability on sports organisations. The recognition of indirect forms of violence and abuse, which is often overlooked due to ignorance, further complicates the challenge of safeguarding athlete wellbeing.^[8]^ Indirect systemic violence and abuse that is enabled, tolerated and accepted within sporting organisations is now under scrutiny.

Initially, the concept of safeguarding was anchored in child protection principles, but it must extend to individuals of all ages, positions and types of athletes, and go beyond regulatory compliance to actively create safe environments where individuals feel secure enough to report abuse openly.^[9]^ Related frameworks and policies guided by the International Olympic Committee have helped create a standard set of actions for sporting organisations to adopt.^[10]^ While there has been an active commitment from sports organisations globally to implement such safeguarding policies and protocols, evidence suggests that athletes, sports medical professionals and related team members may not always be fully aware of their content, scope and responsibilities.^[11]^

Attitudes towards athlete well-being are shaped by cultural and organisational contexts. Findings of Schmidt, Schneeberger and Claussen^[11]^ clearly put forward the complexity of organisational dynamics too. Alas, isolation, imbalanced power structures, normalisation of abusive behaviours, a culture of silence, and a lack of effective reporting channels have enabled abuse patterns to develop and persist in sporting organisations over decades. The mentality of winning at all costs has been associated with increased injury risks and health challenges for athletes. In highly competitive sports environments, implicit pressure on sports medical professionals to prioritise performance outcomes over athlete welfare is common. As a result, athletes (and their physicians) are often caught in an ethical dilemma, facing conflicting priorities further compounded by stigma or fear of retribution.^[13]^ Adoption of the High-Performance Model, which advocates a balance between health and performance, positions medical professionals at the centre of monitoring athletes’ well-being, specifically safeguarding against neglect through overtraining, participation while injured, and exposure to psychological misconduct, amongst others.^[14]^ The integration of artificial intelligence (AI) and data analytics into professional sports has become a gamechanger for athlete injury prevention, recovery, and protection against cyberbullying.^[15]^

Medical and paramedical professionals occupy a unique position within the sports ecosystem, as they routinely interact with athletes and monitor not only their physical well-being, but also their mental state. Their proximity to athletes places them in a critical role for early identification of abuse, neglect and unsafe practices. However, without adequate safeguarding knowledge, these professionals may overlook or misinterpret signs, inadvertently allowing abusive behaviour to continue unchecked. Ensuring requisite understanding of principles, reporting procedures and ethical obligations is essential for upholding accountability within sporting organisations. Also, by conceptualising sports teams as cohesive, integrated units, these approaches emphasis the importance of interpersonal relationships and collective behaviours, offering insight into how certain tendencies and behaviours develop within an organisation. Hence, ensuring that sports medical professionals possess the right attitude towards the advocacy of safeguarding is crucial.

Sporting organisations, with their multidisciplinary teams, have a moral and ethical duty to protect their athletes’ health and well-being. Research by Gillard et al.^[16]^ and Garrod and Rhind^[17]^ highlights that ambiguities often exist regarding role clarity, with safeguarding being a collective rather than a personal responsibility. In this context, medical and paramedical professionals occupy a critical position in which their vigilance and prompt action decisively influence whether interventions succeed or fail. Rugby Africa, as the governing body for the African continent, represents a high-stakes context where safeguarding practices must be robust. Yet advancements in research, policy, and practice are still needed.^[10]^ Assessing the awareness, readiness and confidence of such professionals is therefore essential for gaining insights and identifying gaps that could better guide targeted improvements in policies, training and current practices to help strengthen athlete protection within African Rugby.

## Methods

### Study design and selection criteria

A cross-sectional exploratory study was conducted in Mauritius during the Rugby Africa Men’s 7s competition between June and July 2024. Purposive sampling was employed to recruit ICIR L 2 compliant (Immediate Care in Rugby Level 2) medical or paramedical staff members of the national teams. A total of 25 participants were eligible for inclusion based on the following criteria: medical or paramedical staff (e.g. physicians, physiotherapists, nurses, tournament medical staff) affiliated with the national rugby teams; actively involved in providing medical or paramedical support to athletes before and during the competition.

### Instrument design and data collection

A modified version of the Safeguarding Officer Pulse Survey 2020 was used for this study, which was further consolidated using inputs from the National Olympic Committee (NOC) Survey report from the ‘Building European Safe Sport Together: Assessment of safeguarding status amongst European National Olympic Committees’ project. The instrument relied on four major domains: (i) knowledge concerning safeguarding practices, (ii) identification of misconduct and risk factors within the rugby community, (iii) characterising the severity of misconduct within the sporting field, and (iv) reporting structures and organisational readiness to deal with issues related to the safeguarding of athletes. The questionnaire was reviewed and validated by members of the Safeguarding Framework Group for the Mauritius Rugby Federation, who were mainly physiotherapists, medical staff, and organisation-related staff, and were independent of this study. Given that the instrument was heavily adapted, with items restructured to match the regional context, their input on the contextual nature of the items was deemed relevant, and the feedback collected relied on content validation outputs. The survey consisted of 24 questions. Eligible participants from Zimbabwe, Nigeria, Uganda, Kenya, Algeria, Tunisia, South Africa, Côte d’Ivoire, Burkina Faso, Madagascar, and Mauritius were invited to complete the questionnaire electronically.

### Data analysis

The data was coded and computed using SPSS V21.0. Data was screened and cleaned for missing values resulting from incomplete survey form submissions. Given the descriptive nature of the research, categorical data were either expressed as numbers and percentage. Although composite measures were not computed for items related to environmental risk factors, locus of control for education and awareness, and the organisation’s readiness to manage safeguarding practices, internal consistency measures were conducted to assess their reliability. The following alpha values were recorded: environmental risk factors *α* =0.852 (n=8); education and awareness *α* = 0.874 (n=8), and organisation’s readiness *α* = 0.970 (n=8), where n represents the number of items for each domain.

### Ethical considerations

Informed consent was obtained prior to survey completion. Participation in this study was entirely voluntary and non-remunerated, and participants were free to withdraw at any time without penalty. A possibility of discomfort when discussing safeguarding issues was acknowledged; therefore, to protect anonymity, the countries represented were not disclosed. Data were anonymised and stored securely, accessible only to the research team.

Ethical clearance was obtained by the Ministry of Health & Wellness of Mauritius (reference number MHC/CT/NETH/2025/AV2).

## Results

### Demographic profile of respondents

A total of 19 participants completed the questionnaire, resulting in a 76% response rate. A summary of the demographic profile of respondents is shown in Table 1.

### Awareness of safeguarding policies

Out of the 19 respondents, 10 (53%) answered positively to being aware of rugby-specific safeguarding policies, which were mainly the ‘Safeguarding Policy & Procedures against Harassment and Abuse in Rugby’ (32%) and the ‘Rugby Africa Safeguarding Policy’ (21%). Complementing this perceived low level of awareness was training on those safeguard measures. Only 21% claimed to have completed a safeguarding training course, half of which were refresher courses held every 3 years.

### Safeguarding practices across the medical team

Participants were questioned about their ability to identify signs of abuse or neglect in athletes, to which a majority were fairly confident (63%, 95% CI: 60–65%) versus 11% (95% CI: 9%–12%) who did not feel confident in such practices. Insights regarding the established protocols and procedural pathways that actively follow abuse detection showed that 79% of the medical team personnel would rate themselves from fair to excellent (95% CI: 77%–81%), against 21% (95% CI: 19%–23%), disclosing that they were not familiar with processes within their organisation. The survey revealed a concerning knowledge gap: merely 26% of respondents could confirm the presence of a designated safeguarding officer within their organisation, while a substantial proportion (42%) expressed uncertainty about whether such a critical protection role existed within their team structure at all.

When examining the prevalence of prior experience in abuse cases amongst athletic populations, 84% of the participants responded negatively to having reported a safeguarding concern, while only 11% were satisfied with the corrective measures which were initiated to address the issue. Although concerning behaviours were not observed in their respective organisations (37%), there were still occasions where deviant behaviours were recognised, with psychological abuse and sexual misconduct being the most concerning behaviours (Figure 1).

Participants were aligned with the notion that cases of psychological misconduct (74%, 95% CI: 72%–76%); and physical misconduct (68%; 95% CI: 66%–70%), neglect (68%; 95% CI: 66%–70%), hazing, bullying, cyberbullying (68%; 95% CI: 66%–70%), were serious to reportable concerns (Table 2). While physical misconduct was one of the less common behaviours observed, its occurrence was still reported in some organisations, most commonly as a form of dominance or oppression over the athletes.

In the survey, participants were also provided with case scenarios to assess their understanding of aberrant behaviours and the actions that should be triggered in safeguarding situations. Responses ranged from conducting an investigation and providing support to escalating the concern to senior management or the head of the team (Table 3). An interesting observation was the suggestion of using an experiential process to establish surveillance mechanisms that stimulate a more proactive approach to safeguarding practices.

### Safeguarding practices: Environmental factors and locus of control

Respondents believed education on safe sport practices was instrumental within the organisation (79%, 95% CI: 77%–80%), and that the level of competition could impact athletes’ well-being (68%). Notably, the culture of tolerance for poor practices by higher management (68%, 95% CI: 66%–70%) and fear of repercussions (69%, 95% CI: 67%–71%) were significant contributors to abstaining from disclosure (Table 4). All dimensions constituting environmental factors leaned towards a central location within the rating profile, aligning with a consensus among all determinants regarded as very significant contributors to the safety and well-being of athletes.

Regarding the allocation of responsibility and authority for ensuring athlete protection within competitive sporting and training environments, 89% (95% CI: 88%–90%) of respondents strongly agreed that they were a focal point. This finding advocates the pressing need for education and awareness in the field. This was closely followed by coaches (83%, 95% CI: 81%–85%), the management (78%; 95% CI: 76%–80%); and athletes (68%, 95% CI: 66%–70%) (Figure 2). Interestingly, parents were also believed to play a critical role in the detection of abuse (94%, 95% CI: 93%–95%) within the range of very to extremely important.

### Organisational readiness in handling safeguarding concerns

Respondents were prompted to identify and rate the specific protective measures and safeguarding protocols implemented by their organisations. Measures such as the use of incident report forms, formal interviews, guidelines, and the process for sanctioning adverse behaviours were rated satisfactorily in terms of the organisation’s ability to handle safeguarding concerns (Table 5). However, these findings contradict earlier findings on respondents’ knowledge of organisational disciplinary procedures, in which an overwhelming majority (68%, 95% CI: 66%–70%) reported being unaware of the mechanisms in place. The remaining participants agreed with the process handled by the senior management, based on the findings submitted in the report, and a hearing. This gap signals an incongruity between existing protocols and the methods currently used to address safeguarding issues, strongly suggesting the need to strengthen safeguarding management within the workforce. On a positive note, the readiness and ability to investigate reported cases (53%, 95% CI: 50%–56%) were deemed a gateway to improvements towards more rigid policies and regulations.

## Discussion

Although safeguarding in rugby is in its early stages in most African countries, the capability one should aim for should be sufficient to ensure athletes’ safety in every aspect.^[7]^ However, these efforts are often negatively affected by factors such as inadequate funding, limited resources and infrastructure or low community awareness.^[11,18]^ Educational sessions and sensitisation campaigns often include activities to raise awareness of athlete safeguarding, emphasising both physical and psychological protection; our results demonstrate the continued need for efforts to increase this awareness. Of the 19 respondents, only 10 reported positive awareness of rugby-specific safeguarding policies, with the ‘Safeguarding Policy & Procedures against Harassment and Abuse in Rugby’ (31%) most commonly cited, followed by the ‘Rugby Africa Safeguarding Policy’ (21%). It is important to note that the ‘Rugby Africa Safeguarding Policy’ was nonexistent as of December 2024, while the ‘Safeguarding Policy & Procedures against Harassment and Abuse in Rugby’ is being used by the South African Rugby Union, as of May 2020. This data aligns with numerous researchers’ who call for efforts to increase awareness in this sphere, including health care providers, physicians and families of athletes.^[11,18,19]^

Medical and paramedical staff must be able to recognise, manage, and report harassment or abuse.^[19]^ Yet ignorance, lack of capacity, or refusal to report, along with failure to recognise indirect forms, have contributed to widespread under-reporting. In our study, respondents’ confidence about identifying signs of abuse or neglect in athletes was evident (63%), while those who expressed uncertainty about the same issue were significantly fewer (11%). Such a trend is a favourable sign that the awareness among those in charge is gradually creating a safe sports environment for athletes. Nonetheless, a slightly lower level of appreciation of the deeper mechanisms that follow the disclosure of abuse, and the existence of a safeguarding officer in their union were noted (Table 4). Uncertainty remains over how many athletes and bystanders are unaware of a trusted confidant in abuse situations.^[13]^ Rising risks of sexual, psychological and physical violence along the performance ladder are acknowledged by researchers, hence the need to also strengthen elite athletes’ awareness of reporting unacceptable behaviour. Vertommen et al.^[20]^ reported that 38% of 4,000 minority, disabled, and elite athletes experienced psychological, physical, or sexual abuse before age 18. Similarly, 75% of athletes in UK organised sport were victims of abuse-such findings emphasise further that sports organisations must implement formal governance reforms, with protocols which are better aligned with daily operational practices.

In our study, the fact that 84% of participants responded negatively to having reported a safeguarding issue is suggestive of under-reporting and points to uncertainty about reporting procedures, cultural silence, and fear of retaliation. This should be reframed to acknowledge the likely influence of cultural and organisational barriers.

When we compare this statement with the fact that only 11% of those surveyed were satisfied with the corrective actions initiated to address the issue, we note an incongruence in the information, further pointing to a misalignment between identification and reporting. Our findings are in stark contrast to those reported by Kroshus et al.^[21]^, who found more than 25% experienced pressure from coaches, teammates, parents and fans to continue playing after a concussion. Carroll and Rosner ^[22]^ noted that contact-sport athletes often conceal concussions to avoid career risks, leaving coaches and medical staff in difficult positions to report without firm evidence.^[23]^ This calls for greater awareness and ongoing professional development to navigate such ethical challenges.

Although there was no alarmingly concerning behaviors in the organizations (37%) surveyed in our study, instances of deviant behaviour, inappropriate relationships or psychological misconduct were noted. It is at the top level of performance and elite sport that inappropriate psychological type behaviors are more likely to manifest, as funding for higher level sport is often performance based.^[13,21,23]^ This puts pressure on organisations and coaches to also implement strategies based on an abusive or oppressive approach.^[23]^ Exploring codes of conduct and law enforcement strategies, culturally tailored education for athletes, their parents, sport executives and coaches is proposed as a sensible method of protecting the game. A culture of tolerance of poor practices from higher management and fear of repercussions were identified as significant factors negatively impacting player well-being (Table 3). All the dimensions constituting the environmental factors were leaning towards an approximate mean value of 3.0, which underlines the importance of education in safeguarding and the responsibilities of all involved. De facto, education should not be limited to recognising issues but should also provide information to promote athlete safety and mental well-being. In South Africa, there is an emphasis on the need for formal mental health support programmes for rugby players. Grobler^[24]^ identified the macho image associated with rugby culture as a key barrier to help-seeking behaviour. Hiding injuries reflects the complex interplay between gender, respectability and embodiment of athletic identity. Concealed symptoms can impede athletes’ recovery, potentially cascading downstream and adversely affecting overall team performance.^[23]^ This is where sport medical professionals must step up and be vigilant. Open clear dialogue is a critical aspect of preventing medical mismanagement, neglect and further abuse. Tailored training on how to recognise and respond to cases or suspicions of violence and abuse is essential.^[24]^ Evaluation of the clinical competency of medical and paramedical professions in trauma care and violence recovery is an understudied area that merits further investigation to better understand gaps in the literature regarding the perceptions and practices of these professions in the sporting domain.

In the present study, we report that medical staff agreed that they were the focal point for athlete safeguarding, indicating a recognition of their role. We also highlight the need to provide full assistance to athletes who have been victims of safeguarding breaches. Parents (94%) and coaches (83%) represented the most important positions in the responses, followed by management (78%) and players themselves (68%). Despite this, the responsibility for creating a safe environment for sport does not lie solely within the organisation, but is shared by all those involved. For closure, while mechanisms such as incident reporting forms, formal interviews, and sanctioning processes were rated satisfactory in addressing undesirable behaviour, a notable proportion of respondents (68%) remained unaware of their existence. This discrepancy highlights a gap between policy and awareness, underscoring the need for strengthened communication and education to ensure safeguarding measures in African rugby are both known and effectively implemented. However, given the single-event design, the results of this study may reflect contextual influences such as time constraints, staff turnover, limited opportunities for training, and variability in teams’ prior safeguarding exposure.

### Limitations and suggestions for future research

This study is limited by its small sample size and reliance on self-reported data, which may have introduced response bias and limited the generalisability of findings beyond the Rugby Africa context. The current sample included only participants from a single event involving African countries; therefore, the findings cannot be generalised to sport across the entire African continent, necessitating repeated data collection in similar contexts. Future research should expand to incorporate high-risk athlete perspectives and explore the longitudinal effectiveness of safeguarding training. Further, the development and validation of standardised safeguarding competency measures tailored to medical and paramedical roles in sport is warranted.

## Conclusion

This study has offered insights into the state of safeguarding awareness and practices amongst medical and paramedical professionals within Rugby Africa. Findings highlight a concerning yet actionable gap between institutional safeguarding frameworks and health professionals’ operational knowledge. While a promising proportion of respondents demonstrated confidence in identifying potential abuse, awareness of reporting pathways and organisational protocols remains inconsistent and, at times, critically lacking. These findings highlight a window of opportunity to address knowledge gaps in athlete safeguarding within similar tournament contexts. Preliminary interventions could include introducing mandatory safeguarding training for all staff, clarifying institutional policies and establishing safeguarding officers within unions to strengthen oversight. Standardised reporting pathways may help ensure clinicians focus on athlete support, rather than role-inappropriate investigations. Additionally, raising awareness of monitoring and audit procedures within Rugby Africa could support quality assurance and identify areas for improvement. While these suggestions are relevant for similar competition settings, further cross-national research is necessary before considering broader policy decisions.

## Figures and Tables

**Fig. 1:**
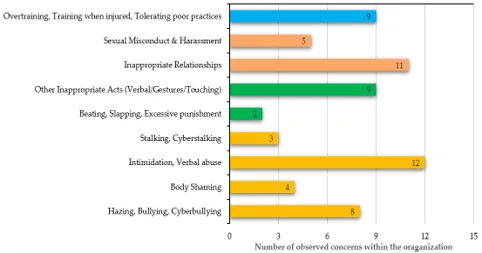
Types of adverse behaviours reported within organisations as part of the safeguarding practices.

**Fig. 2:**
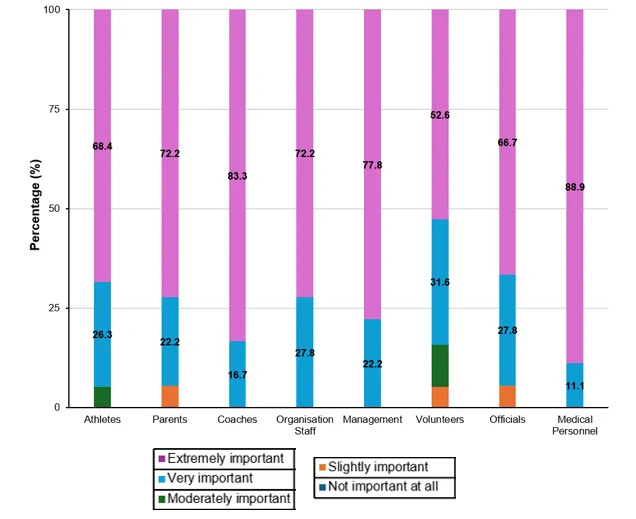
Types of adverse behaviours reported within organisations as part of the safeguarding practices (n=19).

**Table 1:** Demographic profile of respondents

Descriptors		Frequency (n=19)	Percentage (%)
**Age**	**Under 25 years**	1	5.3
	**25–34 years**	14	74
	**35–44 years**	4	21
**Gender**	**Male**	11	58
	**Female**	8	42
	**Team doctor**	2	11
**Team role**	**Physiotherapist**	12	63
	**Nurse**	3	16
	**Tournament medical staff**	2	11
**Years of experience**	**Less than a year**	1	5.3
	**1–3 years**	8	42
	**4–6 years**	2	11
	**7–10 years**	3	16
	**More than 10 years**	5	26

**Table 2:** Frequency of aberrant behaviours observed within sports organisations, categorised in line with the International Olympic Committee safeguarding framework (n=19)

Category	Survey items	Severity of concern			
		Not encountered	Low level of concern	Serious	Reportable
**Psychological abuse / harassment**	Hazing, bullying, cyberbullying	26	5.3	11	58
	Body shaming	21	21	26	32
	Intimidation, verbal abuse	11	16	11	63
**Physical abuse**	Beating, slapping, excessive punishment	26	5.3	11	58
	Other inappropriate acts (verbal/gestures/touching)	21	11	26	42
**Sexual abuse**	Inappropriate relationships (with or without the use of grooming techniques)	32	11	11	47
**Neglect**	Overtraining, training when injured leading to physical harm, tolerating poor practices by others	11	21	26	42

Tabulated data presented as a percentage of the overall respondents. Rating profile, 1 = not encountered, 2 = low level of concern, 3 = serious, and 4 = reportable

**Table 3:** Qualitative inputs concerning measures likely to be implemented in the event of a safeguarding incident

**Scenario 1:** **A player confides in you that they are being bullied by a teammate. What is your immediate course of action?**	Take disciplinary action/ Bring him on a disciplinary board Provide psychological support/reassurance and then report to senior personnel Find out if the information is correct and report it to the coach/manager Listen to the player and establish the facts Talk to the teammate to establish facts To get maximum proof of the accusation and then proceed to report accordingly. To talk to that teammate and to report to the head coach about the issue Report it Speak to management about it Talk to coach Report to the doctor in charge of team Reassure, report to senior Report the information to the head coach Question further to better understand the situation The claims should be taken seriously and the athletes questioned to get to the bottom of the problem Gather the facts, understand the expectations of the victim while reassuring the latter, interview the perpetrator and take the necessary actions. In case of recurrent behavior, harder sanctions should be implemented by the disciplinary committee.
**Scenario 2:** **During a routine check-up, you notice unexplained bruises on an athlete. How do you proceed?**	Try to know how the athlete got the bruises while treating Try and ask where these come from without making it awkward for the athlete and so they can trust me Carry out an interview with the athlete I would privately discuss with the victim to know the cause and find a way to proceed Get his consent and get an assessment done to find out the actual causes of the bruises. Then if in case of abuse follow the reporting process. Try to question these bruises. Find out more information and Report Trying to get the player to speak up about it. Making them check in daily. Close Monitoring Report to coach, medic of team Probe for reasons of bruises and report if anything suspicious Question the athlete, reassure, refer to appropriate professionals, report to senior Investigate cases of abuse Investigate further and report to higher authorities Investigate the source of the bruises, write up a formal report of the incident, separate the victim from the perpetrator if it’s a case of physical abuse, gather the inputs from the perpetrator and assess the severity of the incident before implementing the appropriate sanctions.

**Table 4:** Environmental factors influencing the safety and well-being of athletes (n=19)

Items	NS	SS	MS	VS	ES
Lack of awareness & education on safe sport	0.0	5.3	16	58	21
Young athletes training in a highly competitive environment	5.3	5.3	21	58	11
Overemphasis on winning	11	5.3	21	42	21
Culture of tolerance of poor practices from leaders & management	5.3	11	16	42	26
Culture of fear of speaking up due to perceived consequences	5.3	5.3	5.3	47	32
Poor coaching practices	0.0	11	21	47	16
Poor employment & volunteer screening	0.0	5.3	37	37	16
Lack of safeguarding guidelines & policies	5.3	5.3	16	21	47

Data presented as percentage of overall respondents for the rating profile ranging from 0 = NS (not significant), 1 = SS (slightly significant), 2 = MS (moderately significant), 3 = VS (very significant), and 4 = ES (extremely significant).

**Table 5:** Evaluation of organizational procedures to address reports of safeguarding (n=19)

Checkpoints	Poor	Fair	Good	Very Good	Excellent
Implementation and utilization of incident reporting form	0	26	11	53	11
Interviewing parties involved	0	28	22	44	6
Provision of support for concerned person	0	16	11	68	5
Existence of guidelines/protocols to deal with any breach of code of ethics or safeguard practices	0	26	21	42	11
Investigation and determination of seriousness of case using a guide or framework	0	26	21	42	11
Procedures and guidelines for disciplinary actions	0	21	21	42	16
Implementation of sanction guidelines	0	21	21	42	16
Report writing of the case	0	26	11	42	21

Data presented as percentage of overall respondents for the rating profile ranging from 1 = Poor, 2 = Fair, 3 = Good, 4 = Very good, 5 = Excellent.

## Data Availability

All authors confirm that the data supporting the findings of this study are available within the article.
